# Low HIV drug resistance prevalence among recently diagnosed HIV‐positive men who have sex with men in a setting of high PrEP use

**DOI:** 10.1002/jia2.26308

**Published:** 2024-07-21

**Authors:** Jonathan M King, Francesca Di Giallonardo, Ansari Shaik, Skye McGregor, Julie Yuek Kee Yeung, Tharshini Sivaruban, Frederick J Lee, Philip Cunningham, Dominic E Dwyer, Steven J Nigro, Andrew E Grulich, Anthony D Kelleher

**Affiliations:** ^1^ The Kirby Institute University of New South Wales Sydney Australia; ^2^ New South Wales State Reference Laboratory ‐ HIV/AIDS St Vincent's Hospital Sydney Australia; ^3^ New South Wales Health Pathology‐ICPMR Westmead Hospital Westmead Australia; ^4^ New South Wales Health Pathology‐RPA, Royal Prince Alfred Hospital Camperdown Australia; ^5^ Department of Clinical Immunology and Allergy Royal Prince Alfred Hospital Camperdown Australia; ^6^ Health Protection NSW NSW Ministry of Health Sydney Australia

**Keywords:** ARV, drug resistance, HIV prevention, men who have sex with men, pre‐exposure prophylaxis, treatment

## Abstract

**Introduction:**

New South Wales (NSW) has one of the world's highest uptake rates of HIV pre‐exposure prophylaxis (PrEP). This uptake has been credited with sharp declines in HIV transmission, particularly among Australian‐born gay and bisexual men. Concerns have been raised around the potential for the emergence of tenofovir (TFV) and XTC (lamivudine/emtricitabine) resistance in settings of high PrEP use. Such an emergence could also increase treatment failure and associated clinical outcomes among people living with HIV (PLHIV). Despite low levels of nucleoside reverse‐transcriptase inhibitor (NRTI) resistance relating to PrEP use in clinical settings, there are few published studies describing the prevalence of NRTI resistance among people newly diagnosed with HIV in a setting of high PrEP use.

**Methods:**

Using HIV antiretroviral drug resistance data linked to NSW HIV notifications records of people diagnosed from 1 January 2015 to 31 December 2021 and with HIV attributed to male‐to‐male sex, we described trends in TFV and XTC resistance. Resistance was identified using the Stanford HIV Drug Resistance genotypic resistance interpretation system. To focus on transmitted drug resistance, resistance prevalence estimates were generated using sequences taken less than 3 months post‐HIV diagnosis. These estimates were stratified by timing of sequencing relative to the date of diagnosis, year of sequencing, birthplace, likely place of HIV acquisition, and stage of HIV at diagnosis.

**Results:**

Among 1119 diagnoses linked to HIV genomes sequenced less than 3 months following diagnosis, overall XTC resistance prevalence was 1.3%. Between 2015 and 2021, XTC resistance fluctuated between 0.5% to 2.9% and was 1.0% in 2021. No TFV resistance was found over the study period in any of the sequences analysed. Higher XTC resistance prevalence was observed among people with newly acquired HIV (evidence of HIV acquisition in the 12 months prior to diagnosis; 2.9%, *p* = 0.008).

**Conclusions:**

In this Australian setting, TFV and XTC resistance prevalence in new HIV diagnoses remained low. Our findings offer further evidence for the safe scale‐up of PrEP in high‐income settings, without jeopardizing the treatment of those living with HIV.

## 1 INTRODUCTION

Australia has made remarkable progress in its effort to meet the UNAIDS 2030 target of ending AIDS as a public health threat with HIV diagnoses attributed to male‐to‐male sex declining by 46% between 2013 and 2022 [1, 2]. This decline has largely been ascribed to the high proportion of people living with HIV (PLHIV) with a suppressed HIV viral load and the high uptake of HIV pre‐exposure prophylaxis (PrEP) among gay and bisexual men (GBSM) [1, 3]. In 2022, among GBSM eligible for PrEP recruited in behavioural surveillance surveys, 64% reported using PrEP in the last 6 months, up from around 2% in 2015, one of the world's highest PrEP uptake rates [4, 5].

Concerns have been raised that high levels of PrEP use may be associated with increased HIV antiretroviral therapy (ART) resistance [6]. This ART resistance may occur because of a person either taking PrEP while having undiagnosed HIV (acquired ART resistance) or through acquiring a PrEP‐resistant strain of HIV (transmitted ART resistance) [6]. Indeed, mathematical modelling has demonstrated the potential for an increase in ART resistance associated with widespread PrEP use [7]. However, research has shown that ART resistance associated with PrEP use is minimal when combined with appropriate confirmation of HIV‐negative status prior to PrEP initiation [8].

Oral PrEP, consisting of two nucleoside reverse‐transcriptase inhibitors (NRTI), tenofovir (TFV) in combination with XTC (either lamivudine or emtricitabine), has been included in World Health Organization HIV therapy guidelines since 2013 [9]. Almost all oral PrEP used in Australia consists of TFV (as a disoproxil salt) paired with emtricitabine. To monitor changes in TFV and XTC resistance prevalence associated with increasing PrEP use, UNAIDS has called for HIV antiretroviral resistance surveillance [10]. TFV‐ and XTC‐resistant HIV strains are circulating globally with pretreatment TFV and XTC resistance estimated to be 1.6% and 1.7%, respectively, with regional variations [10]. However, there are few published studies presenting population‐level HIV ART resistance data, including TFV and XTC resistance, in high‐income countries with relatively high PrEP uptake and where the HIV epidemic is concentrated among GBSM. Epidemiological reporting from New South Wales (NSW) and South Australia showed that for 2015 and 2016, overall NRTI resistance prevalence (including non‐XTC/TFV NRTI resistance prevalence) among PLHIV attributed to male‐to‐male sex and diagnosed within the previous 12 months was estimated at 4.3%. A recent study involving people newly diagnosed with HIV in New York City found XTC resistance prevalence, specifically M184I/V mutations, to be 2%, with higher prevalence in those with acute HIV and those who reported recent PrEP use [11].

In Australia, HIV sequence‐based ART resistance testing is recommended at the time of HIV diagnosis [12]. Except for those with a history of PrEP use, any ART resistance identified through this testing is most likely to be transmitted. Drug resistance mutations (DRMs) identified then help inform the optimal ART regimen to avoid treatment failure [11]. In the event of treatment failure any time following ART initiation, additional drug resistance testing is indicated to identify DRMs that may have developed as acquired drug resistance. In NSW, DRM testing is conducted by a limited number of pathology laboratories. As part of this testing, HIV sequence data are compared with the Stanford HIV Resistance Database to identify DRMs.

We aimed to describe changes in TFV and XTC resistance prevalence over time in a setting of high PrEP use among GBSM in NSW, Australia.

## 2 METHODS

DRM records from ART resistance testing are routinely linked to deidentified HIV surveillance data and securely recorded in the NSW HIV Resistance Database, which contains DRM records sampled from 2004 onwards [13]. Multiple sequences may be linked to a single diagnosis record if testing occurs at multiple time points. DRM records linked to HIV diagnoses attributed to male‐to‐male sex and diagnosed between 1 January 2015 and 31 December 2021 were extracted from the NSW HIV Resistance Database. Data fields extracted included date of diagnosis, date of resistance test, age at diagnosis, country of birth, HIV stage at diagnosis, and likely place of acquisition (as reported by the diagnosing clinician). Stages of HIV included newly acquired, not late, late, and advanced. Newly acquired HIV was defined as evidence of HIV acquisition within 12 months of diagnosis, such as a seroconversion illness or negative or indeterminate HIV test within 12 months of diagnosis, irrespective of CD4+ T‐cell count or an AIDS‐defining illness at diagnosis [14]. Not‐late stage was defined as a CD4+ T‐cell count at least 350 cells/µL in the absence of ‘newly acquired’ criteria. Late‐stage was defined as a CD4+ T‐cell count less than 350 cells/µL in the absence of ‘newly acquired’ criteria. Advanced stage was defined as a CD4+ count of less than 200 cells/µL, or an AIDS‐defining illness in the absence of ‘newly acquired’ criteria. Chi‐squared tests were used to identify differences in region of birth, and place of acquisition between HIV diagnoses linked to DRM records and those not linked. An unpaired two‐sample *t*‐test was used to make this comparison by age at diagnosis.

To separate those more likely to have had HIV DRMs prior to diagnosis from those who were more likely to have developed DRMs from receiving HIV ART following diagnosis, HIV sequences were disaggregated into two groups:

Group 1. HIV sequences taken less than 3 months post diagnosis.
Group 2. HIV sequences taken at least 3 months post diagnosis.

Using the Stanford HIV Drug Resistance genotypic resistance interpretation system, HIVdb, identified DRMs were used to assign a level of ART resistance to each sequence, from one (no resistance) to five (high‐level resistance). Sequences were then classified as either ART resistant (resistance levels of four or five) or not ART resistant (resistance levels of three and below). It is possible for a sequence to have more than one DRM, so the DRM conferring the highest resistance level for each drug class (TFV or XTC) was used to classify the sequence. Using the number of sequences analysed as the denominator, DRM resistance prevalence estimates were generated. These estimates were stratified by timing of sequencing relative to the date of diagnosis, year of sequencing, place of birth, likely country of HIV acquisition, and stage of HIV at diagnosis. Differences in DRM prevalence estimates within each stratification were evaluated using Fisher's exact test with *p*‐values < 0.05 considered statistically significant.

Ethical approval for this study was obtained from the NSW Population and Health Services Research Ethics Committee and the ACON Research Ethics Review Committee (RERC) [AU RED Reference: HREC/15/CIHS/38, Cancer Institute NSW reference number: 2015/08/605]. The study data were deidentified prior to use in our study and consent was not sought from those notified with HIV.

## 3 RESULTS

**Figure 1:**
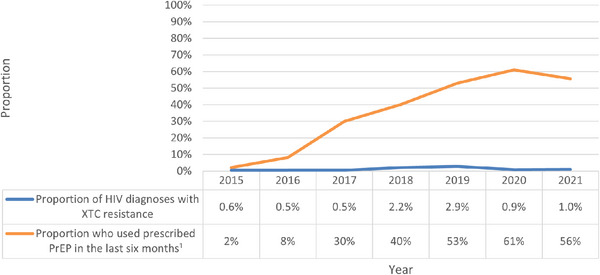
Proportion of new HIV diagnoses with XTC resistance and self‐reported proportion of people suitable for PrEP taking PrEP within the last 6 months, 2015–2021. ^1^ *Data source*: Broady, T., Carna E, Rance J, Lafferty L, Brener L, Treloar C, et al. Annual Report of Trends in Behaviour 2023: HIV/STIs and Sexual Health in Australia. Sydney: Centre for Social Research in Health, UNSW Sydney; 2023; Abbreviations: PrEP, HIV pre‐exposure prophylaxis; XTC, lamivudine/emtricitabine.

Between 2015 and 2021, among 1505 new HIV diagnoses attributed to the male‐to‐male sex in NSW, 74% (1119 diagnoses) were linked to viral sequence data from samples collected less than 3 months following diagnosis. Possible reasons for new diagnoses being unable to be linked to viral genomic data included linkage failure due to missing data, sequencing conducted at a laboratory outside NSW, or a clinician not requesting the sequencing. Among HIV sequences analysed, the median age at diagnosis was 33.0 years (interquartile range (IQR): 27.0–43.0), with 54% born outside Australia, and 68% likely acquiring HIV in Australia. There were no significant differences in age at diagnosis, place of birth, or place of acquisition between notifications linked to HIV sequences and those unable to be linked (data not shown). Overall XTC DRM prevalence was 1.3% (*n* = 14; Table 1). XTC DRM prevalence fluctuated between 0.5% and 2.9% from 2015 to 2021, with a prevalence of 1.0% in 2021 (Figure 1). No TFV resistance was found over the study period in any sequences analysed.

**Table 1:** XTC resistance prevalence among new HIV diagnoses by subcategory, 2015–2021

	XTC‐resistant *n* (%)	Not XTC‐resistant *n* (%)	Total *N*	*p*‐value^a^
Total	14 (1.3%)	1105 (98.8%)	1119	
Age group (years)				
16 to 20	0 (0.0%)	32 (100.0%)	32	0.891
21 to 30	6 (1.5%)	390 (98.5%)	396	
31 to 40	4 (1.2%)	340 (98.8%)	346	
41 and older	4 (1.2%)	343 (98.0%)	352	
HIV stage				
Newly acquired	12 (2.9%)	398 (97.1%)	410	0.008
Not late	1 (0.3%)	303 (99.7%)	304	
Late	0 (0.0%)	168 (100.0%)	168	
Advanced	1 (0.4%)	233 (99.6%)	234	
Not reported	0 (0.3%)	3 (100.0%)	3	
Place of birth				
Australia	8 (1.5%)	510 (98.5%)	518	0.433
Outside Australia	6 (1.0%)	594 (99.0%)	600	
Not reported	0 (0.0%)	1 (100.0%)	1	
Likely place of acquisition				
Australia	13 (1.7%)	749 (98.3%)	762	0.078
Outside Australia	1 (0.3%)	319 (99.7%)	320	
Not reported	0 (0.0%)	37 (100.0%)	37	
Year of sequencing				
2015	1 (0.6%)	173 (99.4%)	174	
2016	1 (0.5%)	192 (99.5%)	193	
2017	1 (0.5%)	189 (99.5%)	187	
2018	4 (2.2%)	178 (97.8%)	182	
2019	5 (2.9%)	170 (97.1%)	175	
2020	1 (0.9%)	105 (99.1%)	106	
2021	1 (1.0%)	101 (99.0%)	102	0.358

^a^ Calculated using Fisher's exact test, tests excluded *Not reported* in each subcategory; Abbreviations: XTC, lamivudine/emtricitabine.

By age group, XTC resistance prevalence was found to be 1.5% among those aged 21 to 30 years (*n* = 6), 1.2% among those aged 31 to 40 years (*n* = 4), 1.2% among those aged 41 years or older (*n* = 4), with no instances observed among those aged 16 to 20 years (*p* = 0.29). By stage of HIV, a greater XTC resistance prevalence of 2.9% was observed among individuals diagnosed with newly acquired HIV, compared to other stages of diagnosis (*p* < 0.01; Table 1). Similar XTC prevalence rates were observed among individuals born in Australia (1.5%) and those born outside Australia (1.0%), with no statistically significant difference noted (*p* = 0.43). Among those likely to have acquired HIV in Australia, a higher XTC prevalence of 1.7% was observed, compared to those likely acquiring HIV outside Australia (0.3%), although this difference was not statistically significant (*p* = 0.08). Among people who were diagnosed with newly acquired HIV, 78% were likely to have acquired HIV in Australia (data not shown).

By specific DRM, of sequences with XTC resistance taken less than 3 months after diagnosis, M184V/MV/MI/MIV DRMs (*n* = 14) were observed. In combination with these DRMs, two other DRMs were observed, including, S68N (*n* = 1) and S78G (*n* = 1).

From 88 sequences taken 3 months or more after diagnosis, observed XTC resistance prevalence was 6.8% (*n* = 6) with no TFV resistance observed (data not shown). Of sequences with XTC resistance, M184V/I (*n* = 6) was observed as well as K70E (*n* = 1).

## 4 DISCUSSION

Despite enormous increases in PrEP uptake among GBSM living in NSW between 2015 and 2021, XTC resistance prevalence remained low, and TFV resistance was not observed during the study period. This finding offers further evidence that in settings of high PrEP uptake, the risk of increased DRM prevalence remains low and that increasing PrEP availability will not compromise the effectiveness of ART for PLHIV, particularly for those on integrase strand transfer inhibitor‐based ART [15].

The prevalence of XTC and TFV resistance among sequences sampled within 3 months of diagnosis was lower than previous regional pre‐treatment NRTI resistance prevalence estimates among GBSM in the Americas, Europe, Southeast Asia and the Western Pacific [7]. The estimates presented here are lower than those previously reported for NSW and South Australia, likely because these prior estimates were based on overall NRTI prevalence rather than being specific to XTC prevalence. Similar XTC resistance prevalence was recently reported among people with a new HIV diagnosis using data from surveillance and partner services in New York City [11]. The lower TFV resistance prevalence, compared to XTC prevalence, is consistent with the tendency for XTC resistance to develop more readily than TFV resistance [16].

By stage of HIV at diagnosis, greater XTC resistance prevalence was observed among people diagnosed with newly acquired HIV. This finding is consistent with previous studies demonstrating a greater number of M184V DRMs among people with early‐stage HIV due to a tendency of this DRM to revert to ‘wild‐type’ over time in the absence of XTC‐containing treatment [11, 17, 18]. This tendency suggests that the higher prevalence of XTC resistance observed among people diagnosed with newly acquired HIV was more likely due to transmitted ART resistance rather than to PrEP use while living with undiagnosed HIV. Further, the inference that most XTC resistance may have been transmitted also suggests meaningful levels of XTC resistance among PLHIV on ART. Therefore, our findings support a three‐drug regimen as the first‐line standard regimen to limit treatment failures attributed to XTC resistance.

Although not statistically significant, nearly all sequences exhibiting XTC resistance were from individuals likely acquiring HIV in Australia. This higher prevalence may relate to the fact that people who acquire HIV abroad are less likely to receive an early diagnosis and therefore less likely to present with transmitted ART resistance. Indeed, in our study, more than three‐quarters of people who were diagnosed with newly acquired HIV likely acquired HIV in Australia. However, it should be acknowledged that the clinician estimate for the likely place of acquisition may be subject to interviewer and/or recall bias and the DRM prevalence estimate should be interpreted with caution [19].

ART resistance was not available for around a quarter of new diagnoses and our findings may not be fully representative of all new HIV diagnoses. However, there was little difference between the sociodemographic characteristics of new diagnoses linked to HIV sequences and those unable to be linked, suggesting that the risk of bias is low. ART resistance testing at the time of HIV diagnosis remains an important part of HIV management.

It is possible that DRM prevalence between 2020 and 2021 may have been influenced by the Coronavirus pandemic, impacting both PrEP use and the proportion of HIV diagnoses tested for ART resistance. However, according to Pharmaceutical Benefits Scheme data, the number of people prescribed PrEP within the past 12 months in NSW increased by 24% between 2019 and 2021 [20]. Still, the low number of DRMs identified across the entire study period means that trends over time should be interpreted with caution.

The higher DRM prevalence among those who had DRM testing undertaken 3 months or more following treatment may relate to the fact such people are more likely to have HIV DRM testing due to treatment failure rather than routine screening. Therefore, the DRM prevalence among this group is unlikely to represent the wider population of PLHIV.

## 5 CONCLUSION

Despite concerns that high levels of PrEP uptake may lead to associated increases in HIV ART resistance, our study has shown that in a location with one of the world's highest PrEP uptake rates, TFV and XTC resistance prevalence remains low. Our findings offer further evidence for the safe scale‐up of the global PrEP rollout, without jeopardizing the treatment of those living with HIV.

## COMPETING INTERESTS

The authors have no competing interests.

## AUTHORS’ CONTRIBUTIONS

Conceptualization: FDG, AG and ADK. Data curation: FDG, AS and SN. Sequence data: JYKY, TS, PC, DED, FJL. Demographic data: SN. Funding acquisition: ADK and AG. Methodology: JK and FDG. Supervision: ADK and AG. Visualization: JK. Writing—original draft: JK and FDG. All authors have read and approved the manuscript.

## Data Availability

ADK and FDG were funded by the Medical Research Future Fund (MRFF) Genomics Health Futures Mission—Pathogens Genomics Grant Opportunity (FSPGN000047). The Kirby Institute also receives funding from the Australian Government Department of Health and Aged Care.
